# Effects of COVID-19 vaccine safety framing on parental reactions

**DOI:** 10.1371/journal.pone.0302233

**Published:** 2024-04-16

**Authors:** Hao Tan, Jiayan Liu, Yingli Zhang

**Affiliations:** 1 Lushan Lab, Hunan University, Changsha, China; 2 School of Design, Hunan University, Changsha, China; Regional Health Care and Social Agency of Lodi, ITALY

## Abstract

As a major concern shared by parents globally, COVID-19 vaccine safety is typically being messaged to the public in a negative frame in many countries. However, whether the COVID-19 vaccine safety framing have an effect on parents when vaccinating their children is unclear. Here we implement an online survey with a convenience sample of 3,861 parents living in mainland China, all over 18 years old and with at least one child under 18. The parents were randomly assigned to receive information about COVID-19 vaccine safety in either a negative frame (incidence of side effects) or a positive frame (the inverse incidence of side effects), to compare parental reactions to a range of questions about communication, risk perception, trust, involvement and behavioral intention. We found that parents were more likely to regard vaccine safety as relevant to policy support and as a higher priority for government when receiving positively framed information (p = 0.002). For some specific subgroups, parents in positive framing group showed lower risk perception and higher trust (p<0.05). This suggests that positive framing of COVID-19 vaccine safety messages show more effective performance than negative framing in terms of involvement, as well as trust and risk perception in specific subgroups, which may lead to a reflection on whether to adjust the current widespread use of negative framing. Our findings inform how governments and health care workers strategically choose the framing design of COVID-19 vaccine safety information, and have important implications for promoting COVID-19 vaccination in children in the future.

## Introduction

Despite the benefits and worldwide approval use of COVID-19 vaccination for children [1–7], parents still remain a high level of vaccine hesitancy due to concerns about vaccine safety [8–15]. Growing evidence showing that behavioral nudges, which are usually shifts in how a message is framed, are desperately needed to boost COVID-19 vaccination [16–20]. However, public-health specialists and healthcare workers face a particular dilemma in communicating vaccine safety information to parents [21], because they are not provided with guidelines for presenting or framing the information. Public health agencies in many countries such as China, the UK and the US are using a negative frame (incidence of side effects) when explaining COVID-19 vaccine safety [22–26], but our understanding of the framing effect is limited. Understanding how the framing of COVID-19 vaccine safety information affects parental reactions helps examine the appropriateness of the frame currently used in most countries, and may help to address the challenge of risk communication regarding COVID-19 vaccination in the future when China rolls out COVID-19 vaccine for younger children.

Human choices and attitudes are influenced by the manner in which information are presented, referred to as framing effects [27, 28], and such insights are beginning to be applied in the field of vaccination. Attribute framing, which manipulates an object’s quality or characteristics in a positive or negative frame [29], has been examined in many studies [30–32], and has been shown that subtle changes in the framing may have meaningful effects on readers’ understanding and reactions to information.

In previous research, scholars mainly focused on how different frames can affect participants’ risk perception or behavioral intention. Most of these studies found that positive framing led to significantly lower risk perceptions [33, 34] and significantly higher behavioral intentions [26, 35, 36], but some studies did not demonstrate a significant effect on either risk perceptions or behavioral intentions [37]. Therefore, we hypothesized that compared with parents who received negatively framed information about COVID-19 vaccine safety, parents who received positively framed information had a significantly lower perceived risk of COVID-19 vaccine side effects (hypothesis 1) and a significantly higher intention to vaccinate their child when the vaccine was available (hypothesis 2). As evolving evidence suggests that regular or seasonal booster vaccinations against COVID-19 may be necessary [38], we also examined parents’ intentions to vaccinate their children regularly in the future. We hypothesized that parents in the positive framing group would show a higher intention to get their children vaccinated regularly (hypothesis 3).

Communication and trust are also issues that are often explored in attribute framing studies. Research evidence suggests that positively framed statements were more appealing to transmit [39], so we hypothesized that parents in the positive framing group were more likely to share vaccine safety information with family and friends (hypothesis 4). In a classic study of attribute framing, negative framing was found to weigh more in trust assessments [40], whereas Webster and Rubin showed no difference in the performance of trust between the two frames [34]. Therefore, we hypothesized that parents in the negative framing group would be significantly more trusting of the government’s reporting of information on the safety of the COVID-19 vaccine (hypothesis 5).

In addition, we also considered the effects of framing on involvement, including policy support and perceptions towards government priorities. Policy support is an important topic in framing studies, particularly in the field of environment and climate [41–44]. Results from attribute framing studies on vaccines suggested that participants exposed to positive framing would be more supportive of vaccine policy than those exposed to negative framing [45], so we hypothesized that parents in the positive framing group are more likely to be involved in vaccine policy support (hypothesis 6). The issue of judgments on government priorities has not been explored extensively and deeply in framing research, although a study on goal framing allowed participants to prioritized a list of potential government actions to test framing effects [46]. We hypothesized that parents in the positive framing group would give vaccine safety a significantly higher level of government priority (hypothesis 7).

In terms of framing research on COVID-19 vaccines, most studies have explored the effects of the goal framing by emphasizing the benefits of vaccinating or the losses of not vaccinating [47–58], and some studies have examined framing effects by emphasizing other conditions (e.g. individual-centered versus collective-centered) [59–62]. Only a few studies have focused on the message of COVID-19 vaccine safety, and investigated whether the framing of vaccine safety information has effects on the public reactions [26, 35, 63].

Although findings have shown that COVID-19 vaccine safety can influence vaccination of children [64–66], there is still insufficient research focusing on the effects of the COVID-19 vaccine safety framing on parents. Considering the importance of COVID-19 vaccine safety communication and the differences in its application to populations with different characteristics [67], it is essential to understand parental reactions to the COVID-19 vaccine safety framing and to present accurate vaccine safety information in an understandable and convincing form.

Using the Chinese parents as an example, this study compared the framing effects between the COVID-19 vaccine safety information in positive frame (the inverse adverse event rate) and negative frame (the adverse event rate) on multiple dimensions of COVID-19 vaccine belief and behavioral intentions. Specifically, we explored whether parents who received positively-framed information about COVID-19 vaccine safety were more likely to share vaccine safety information, support vaccine policies, and give vaccine safety a higher level of government priority, and exhibited higher intention to get their child vaccinated when the vaccine was available and to vaccine them on regular, while showed lower risk perception and trust. We also added sociodemographic characteristics and baseline COVID-19 vaccine mood as covariates for analysis and considered whether there was an interaction between framing and them. Finally, in addition to examining the effect of framing in the general parent population, we divided the sample according to socio-demographic factors and conducted subgroup analyses.

The remainder of this study is structured as follows: the “Results” section explains the t-test results, MANCOVA results, ANCOVA results and subgroup analysis results; the “Discussion” section offers a discussion of the empirical findings, practical implications, limitations, research contributions and protentional future research suggestions; and the “Materials and Methods” section provides details of the sample, experiment design, measurements and data analysis.

## Methods

### Ethics statement

This study was approved by the Research Ethics Committee of Hunan University (2019002). Written consent was obtained from respondents when they registered and completed the questionnaire on the online survey platform, and they were assured that all results would be disseminated in aggregate form to guarantee anonymity and confidentiality.

### Data

Our sample was derived from an online survey conducted across China from 18 January to 1 February 2022, that targeted Chinese parents whose children are under 18 years old. The survey was performed on Sojump (www.sojump.com), a professional online survey network with 52,000,000 users in China. Convenience sampling approach was adopted to recruit participants from the online survey network, which is an appropriate non-probability sampling method for researchers who need to recruit participants that meet specific criteria and widely used in exploratory research [68]. Furthermore, previous framing effect studies have effectively utilised convenience sampling [69, 70]. Eligible parents had to be at least 18 years old, living in mainland China and at least one child under 18 years of age. Participants who complete the online survey will receive 120 rewards points from the survey company to redeem for money.

**Fig 1:**
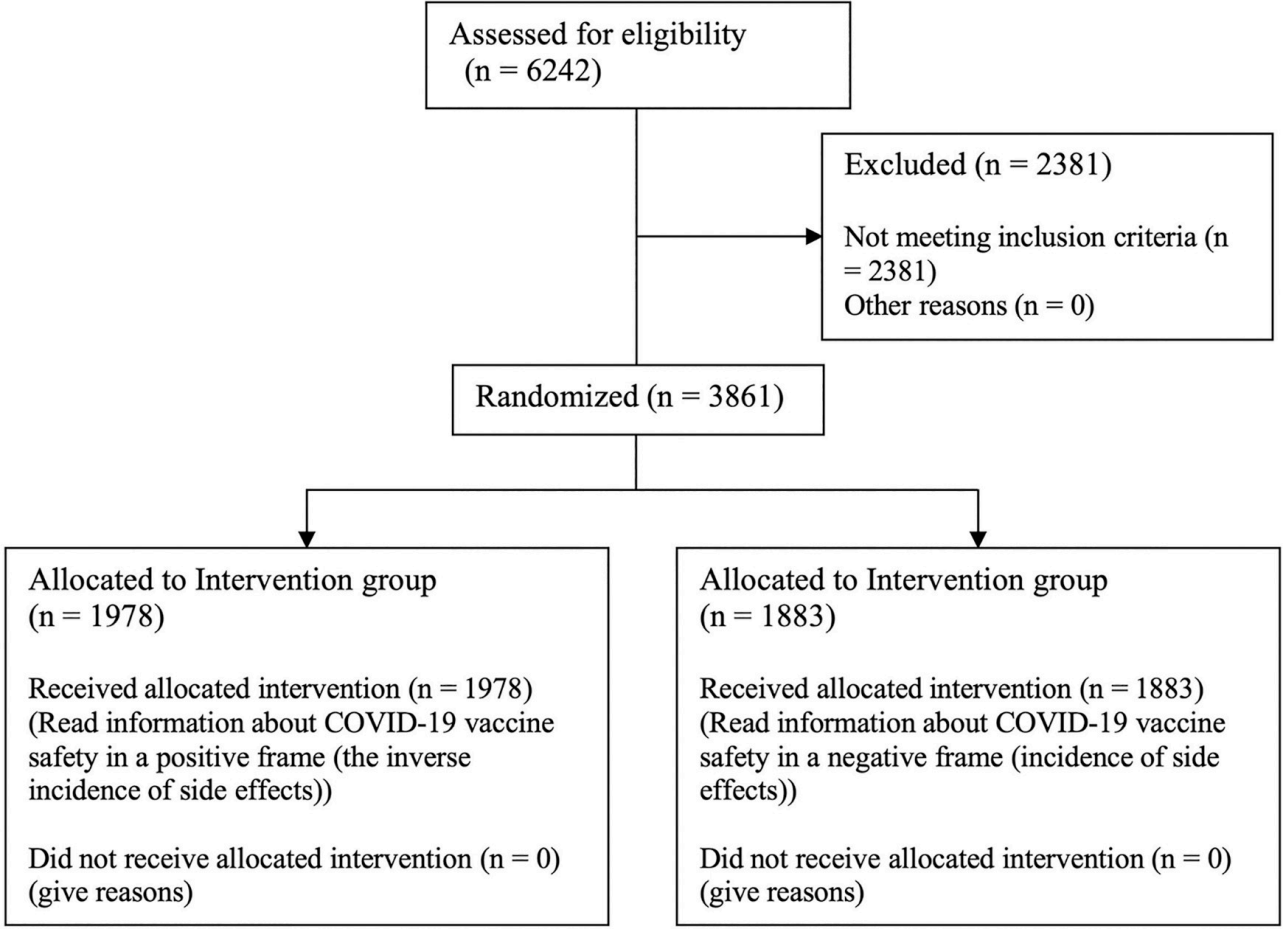
CONSORT flow chart.

Among the 3861 participants, 1978 (51.2%) received COVID-19 vaccine information in the positive frame (here after ‘positively-framed information’ sample) and the remaining participants (1883, 48.8%) were asked questions after receiving COVID-19 vaccine information in the negative frame (here after ‘negatively-framed information’ sample) (Fig 1). No differences were observed between the positively-framed information sample and the negatively-framed information in terms of gender distribution, age structure, education distribution, distribution of parents’ COVID-19 vaccination status, or distribution of their children’s influenza vaccination status.

### Survey experiment design

The experiment began with the following question wording: ‘Here are a couple details about the COVID-19 vaccine, which was confirmed by experts in the immunization program of the Chinese Center for Disease Control and Prevention:’. In one treated group, respondents who received positively-framed information were told: “In every dose of COVID-19 vaccine, there is a more than 99.988% chance that an adverse event will not occur in vaccinated population. (According to the results of the analysis as of 30 April 2021).” Respondents in the other treated group who receiving negatively-framed information were told: “In every dose of COVID-19 vaccine, there is a less than 0.012% chance that an adverse event will occur in vaccinated populations. (According to the results of the analysis as of 30 April 2021).” And both of them were told that according to the current monitoring analysis, the probability of vaccine side effects in children and adolescents was no higher than that in adults over the age of 18. Then, all participants were asked the same questions in the same order, except for random assignment to a baseline of vaccine safety information.

### Measures

Parental reaction indicators include communication, belief (risk perception and trust), involvement (policy support and government priority judgement) and behavioral intention (vaccination when available and on regular basis). The survey questions were adopted or revised versions of questions from relevant studies [26, 35–37, 39, 45, 71–73].

#### Communication

We measured communication using two items with each scored on a 5-point scale (1  =  ‘very unlikely’ to 5  =  ‘very likely’). Items include, ‘How likely would you be to talk to your family members about issues related to COVID-19 vaccine side effects with your family members?’, and ‘How likely would you be to talk to your friends about issues related to COVID-19 vaccine side effects with your friends?’. The questions were revised from relevant studies [39, 45], and the internal reliability was excellent (α  =  0.812).

#### Risk perception

Three items assessing parents’ risk perception were taken from the Renner’s study of A/H1N1 influenza vaccination [71]. These were, (1) ‘How severely do you think the COVID-19 vaccine side effects could have harmed your children?’ (scored on a 5-point scale ranging from 1 ‘not serious’ to 5 ‘very serious’), and, (2) ‘How worried would you be about the impact of the COVID-19 vaccine side effects?’ (scored on a 5-point scale ranging from 1 ‘not worried’ to 5 ‘very worried’), and, (3) ‘How likely would your children be to experience an adverse event with COVID-19 vaccine?’ (scored on a 5-point scale ranging from 1 ‘very unlikely’ to 5 ‘very likely’). The internal reliability for this variable was excellent (α  =  0.758).

#### Trust

To measure parents’ trust, they were asked whether they believe that the CDC is faithfully reporting the risks of the COVID-19 vaccine [36, 72], and responses were recorded on a 5-point scale from 1 ‘do not trust at all’ to 5 ‘completely trust’.

#### Involvement

Similar to a climate change labelling effects research [73], we measured the question of involvement. Participants were asked, ‘Do you agree that the issue of an adverse event with COVID-19 vaccine is an important consideration for your decision regarding whether support COVID-19 vaccination policies for children?’ (scored on a 5-point scale ranging from 1 ‘strongly disagree’ to 5 ‘strongly agree’). Participants were also asked, ‘Do you think that safety of COVID-19 vaccines should be a low, medium, high, or very high priority for the government?’, and responses were recorded on a four-point Likert scale (1  =  low, 2  =  medium, 3  =  high, 4  = very high).

#### Behavioral intention

Participants rate their likelihood of questions “How likely would you be to get your children a COVID-19 vaccine after the vaccine becomes available?” and “If regular COVID-19 vaccination is needed, how likely would you be to get your children vaccinated on a fairly regular basis?” Response options ranged from ‘very unlikely’ (1) to ‘very likely’ (5) with higher scores reflecting higher levels of vaccination intention [26, 35–37].

To measure basic mood about COVID-19 vaccine, participants were asked, ‘How did you feel about COVID-19 vaccine?’ with response scale from 0 (‘extremely bad’) to 100 (‘extremely good’) [74]. We also collected information on gender (male and female), children’s age (under 3 years, 3–11 years and above 11 years), educational attainment (high school and below, junior college, undergraduate and postgraduate and above), and income (low, middle and high income). The Chinese-English translation was done by the second author and was reviewed by survey experts from the Sojump Research Website.

### Analysis

The survey data were managed using SPSS. We used two-sided independent-samples t-tests to examine the framing effects between the two groups. Table 1 provides the results of the t-tests that examined the framing effects on the full data set. We compared the parental reactions of different information framings using a multivariate analysis of covariance (covariates: parents’ gender, education, income, children age and basic mood for COVID-19 vaccine). Following the MANCOVA models, we also ran separate univariate analyses of covariance (ANCOVA) to test which of the dependent variables were statistically significant.

**Table 1:** Two-sided independent sample t-test results of framing effects for the whole sample.

Construct	Frame	N	Mean (s.d.)	*p*	Effect size (r)
Communication	positive	1978	3.30 (1.096)	0.582	0.009
	negative	1883	3.28 (1.086)		
Risk perception	positive	1978	2.41 (0.950)	0.728	0.006
	negative	1883	2.42 (0.959)		
Trust	positive	1978	4.10 (0.896)	0.108	0.026
	negative	1883	4.06 (0.912)		
Involvement—policy support	positive	1978	3.85 (1.022)	0.058	0.031
	negative	1883	3.79 (1.034)		
Involvement—government priorities	positive	1978	3.38 (0.720)	0.002	0.050
	negative	1883	3.30 (0.745)		
Behavioral intention—when available	positive	1978	4.28 (0.942)	0.536	0.010
	negative	1883	4.26 (0.930)		
Behavioral intention—regular vaccination	positive	1978	4.13 (0.975)	0.237	0.019
	negative	1883	4.09 (1.006)		

The Box M test confirmed the homogeneity of the variance–covariance matrices. And we also checked the interaction between framing and covariates, which were not statistically significant (Wilk’s Lambda measures of 0.998–0.999, p > 0.05). The 7 dependent variables were not normally distributed; however, since our sample size was sufficient to obtain robust results (n > 1000 in both groups), we followed the standard parametric procedure for the univariate analysis.

In addition to examining the framing effects of the general parent population, we divided the sample according to several key characteristics, which are central to the discussion of parental reactions, as follows: parents’ gender, education, income and children age. Differences among these groups were tested by t-test, which could improve understanding of how sociodemographic factors influence framing effects.

As discussed above, we were aware that the 7 outcome variables were not normally distributed. However, the t-test has been found to be robust when data are non-normally distributed, particularly with a large sample size [75, 76], so we kept the parametric method in our analysis.

Nevertheless, we performed Mann–Whitney tests for all analyses. We found no difference for full sample, and found that statistical significance was different in one case of subgroup analysis, between the two tests. The case was policy support for parents whose children were 3–11 years old, where in t-test it was statistically significant (p = 0.033, r = 0.048), but in Mann–Whitney test it was not significant (asymptotic significance p = 0.329, r = -0.019).

Statistical analysis was performed using SPSS. The statistical significance level was established at p < 0.05, however, we also reported a marginally significant effect at 0.06 > p > 0.05.

## Results

Our data were derived from a large online survey (N = 3861) conducted across China in 2022, during the period when children were receiving the COVID-19 vaccine. We first performed a series of two-sided independent samples t-tests to identify differences in framing effects between the negative frame sample and positive frame sample for the full sample. The 7 dependent variables were not normally distributed; however, the t-test has been found to be robust when data are non-normally distributed, particularly with a large sample size. We also performed the Mann-Whitney test and found no significant difference between two tests.

**Fig 2:**
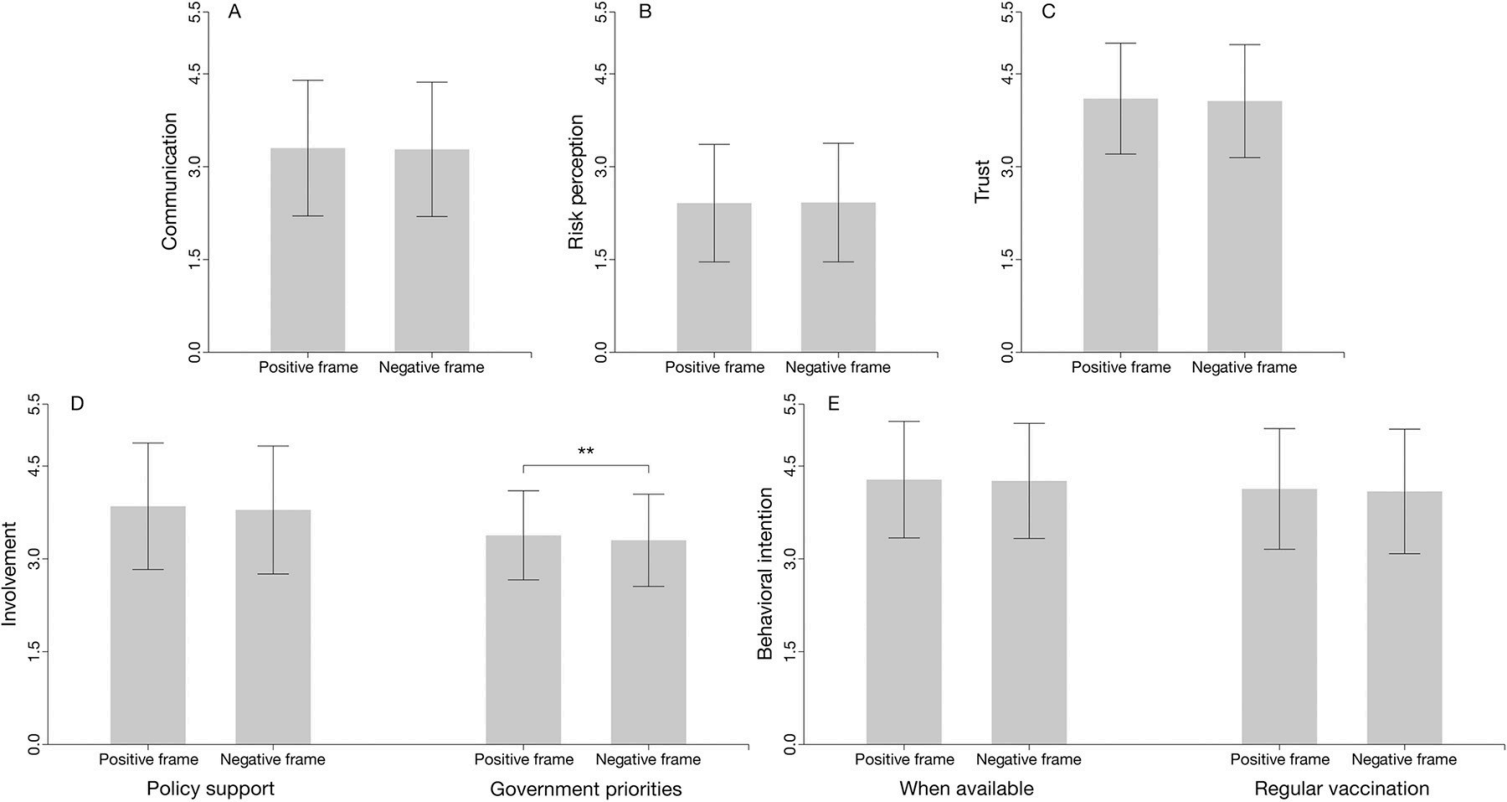
Framing effects for the whole sample, as corresponding to those shown in Table 1.

Significant difference in the involvement (government priority perception), and marginally significant difference in the involvement (policy support) were found between framing groups (Table 1; Fig 2). Parents in negative framing group rated significantly lower level in priority for governments (negative frame M = 3.30 vs. positive frame M = 3.38, p = 0.002). Also, parents were less likely to perceive vaccine safety as relevant to vaccine policy support when receiving negatively-framed information than when receiving positively-framed information (negative frame M = 3.79 vs. positive frame M = 3.85, p = 0.058). This suggests that Chinese parents’ reactions to involvement were generally influenced by the COVID-19 vaccine safety information framing effect. However, no significant differences were found in communication, belief and behavioral intention (all p > 0.1, Table 1).

The MANCOVA model show that the differences between positive and negative frames in parental reactions (Wilks λ  =  0.996, F (7,3848)  =  2.027, p  =  0.048) remained significant after controlling for parental gender, education, income, children age and basic mood of vaccine (Table 2).

**Table 2:** Results of the MANCOVA model of associations between framing, gender, education, income, basic mood, children age and parental reactions.

Model	Wilks λ	F	*p*	Effect size (η^2^)
Intercept	0.445	685.63	< 0.001	0.555
Framing	0.996	2.03	0.048	0.004
Gender	0.988	6.88	< 0.001	0.012
Education	0.989	6.17	< 0.001	0.011
Income	0.989	6.12	< 0.001	0.011
Children age	0.993	3.82	< 0.001	0.007
Mood	0.847	99.05	< 0.001	0.153

Similar with the results of t-test, The results of the univariate analysis show a significant association between information framing and public support (F (1,3854)  =  4.06, p  =  0.044, η2  =  0.001) as well as government priority perception (F (1,3854)  =  10.275, p  =  0.001, η2  =  0.003). The full result of univariate analysis of MANCOVA models for parental reactions are reported in supplementary information.

We then considered the framing effects for sociodemographic factors. The results showed no difference in framing effects between the two frames in parents with children under three years of age, with low (high school and below degree) or high (postgraduate and above degree) education and high income level.

In contrast, several issues showed significant differences in parents with gender, children above three years of age, middle education level, and lower income levels (Table 3; the full results are reported in supplementary document).

**Table 3:** Two-sided independent sample t-test results of framing effects for subgroup.

Construct	Frame	N	Mean (s.d.)	*p*	Effect size (r)
**Gender: male**					
Involvement—government priorities	positive	746	3.48 (0.707)	0.035	0.056
	negative	694	3.40 (0.747)		
**Gender: female**					
Trust	positive	1232	4.09 (0.880)	0.038	0.042
	negative	1189	4.02 (0.931)		
Involvement—government priorities	positive	1232	3.31 (0.721)	0.022	0.046
	negative	1189	3.25 (0.739)		
**Educational: junior college degree**					
Trust	positive	228	4.18 (0.878)	0.042	0.097
	negative	208	4.00 (0.922)		
**Educational: undergraduate degree**					
Involvement—government priorities	positive	1502	3.38 (0.709)	0.007	0.049
	negative	1458	3.31 (0.725)		
**Income: low income level**					
Trust	positive	430	4.09 (0.850)	0.016	0.084
	negative	389	3.94 (0.935)		
Involvement—government priorities	positive	430	3.30 (0.734)	0.041	0.072
	negative	389	3.20 (0.756)		
**Income: middle income level**					
Involvement—government priorities	positive	1043	3.38 (0.714)	0.010	0.057
	negative	1032	3.30 (0.753)		
**Children age: 3–11 years old**					
Involvement—policy support	positive	1201	3.84 (1.027)	0.033	0.044
	negative	1136	3.74 (1.073)		
Involvement—government priorities	positive	1201	3.38 (0.713)	0.020	0.048
	negative	1136	3.31 (0.732)		
**Children age: above 11 years old**					
Risk perception	positive	245	2.24 (0.960)	0.044	0.091
	negative	254	2.42 (1.009)		

In addition to involvement, we observed framing effects on parental trust and risk perception in some subgroups.

Specifically, Female respondents displayed less trust in the COVID-19 vaccine safety information when the negatively-framed information was received than when the positively-framed information was received (negative frame M = 4.02 vs. positive frame M = 4.09, p = 0.038), and the same effects were observed among parents with a junior college degree (negative frame M = 4.00 vs. positive frame M = 4.18, p = 0.042) and low income level (negative frame M = 3.94 vs. positive frame M = 4.09, p = 0.016).

Moreover, when receiving the negatively-framed information, parents with children above eleven years of age had a significantly higher risk perception than when receiving the positively-framed information (negative frame M = 2.42 vs. positive frame M = 2.24, p = 0.044).

## Discussion

Similar to some previous studies showing that subtle changes in framing had an impact on people’s reactions [45, 77, 78], our study revealed a significant framing effect for parents’ involvements. Specifically, parents exposed to positively framed COVID-19 vaccine safety messages were more likely to regard vaccine safety as relevant to policy support and as a higher priority for government than parents exposed to the same messages in the negative frame. In addition, more framing effects on trust and risk perception were observed among female participants, parents with children aged 11 years or older, parents with a junior college degree, and those on low incomes. The results suggest that the negative framing of COVID-19 vaccine safety information, which is widely used worldwide, should be used with particular caution, and that health professionals and policy makers need to carefully consider how to present information well.

The finding that parents were more likely to involve in vaccine policy support when receiving COVID-19 vaccine safety information in the positive frame than in the negative frame, are consistent with a study on human papillomavirus (HPV) vaccine that respondents exposed to positive framing were more supportive of vaccine mandate policy [45].

In addition, our research showed that presenting COVID-19 vaccine safety information to parents in the positive frame improved their perception of the government’s priorities on vaccine safety issues, more than presenting the same information in the negative frame. Based on a previous study on climate change showing that the respondents’ perceived susceptibility had a positive effect on the attitude towards government’s priority [79], the positively-framed information may lead to higher parental perceived susceptibility of COVID-19 vaccine side effects and therefore they believe that the government should give high priority to the safety of COVID-19 vaccines.

For framing effects in subgroup analysis, many backlash effects were observed. One backlash effect we found was that those mothers, parents with a junior college degree and low income level were less likely to trust the CDC-reported COVID-19 vaccine safety information when the negatively-framed information was received than when the positively-framed information was received.

This finding is different from the result observed for ground beef advertisement [80], which revealed negative frames are more influential for establishing trust. One possibility for the different results is that the research on ground beef advertising explored trust not in official government agencies but in individuals engaged in certain professions, such as merchants whose interests were perceived to be diametrically opposed to those of their clients. Thus, identifying the causes for this reaction requires further investigation.

We also found that compared with parents who received positively framed information about COVID-19 vaccine safety, those with children aged 11 years or older had significantly higher perceived risk of COVID-19 vaccine side effects when receiving negatively framed information. We have observed the impact of framing effects on risk perception in many studies [33, 34], but explaining the reasons for different reactions towards perceived risk of COVID-19 vaccine among parents of adolescents under the framing effect needs further study.

Overall, our results revealed a significant framing effect on parents’ involvements, which would play an important role in policy development [81, 82]. Also, we identified many negative, backlash framing effects for some specific subgroups, such as mothers. Understanding the framing effects for these groups are crucial to targeting audiences COVID-19 vaccine risk communication.

The current reporting of official data on vaccine safety information in many countries is based on negative framing of adverse event rates, and our study found negative framing effects in some specific populations under this frame. Our findings inform that the currently widely used negative framing needs to be seriously reconsidered, and we hope to provide public-health specialists and healthcare workers with some guidelines for presenting or framing the information when communicating the COVID-19 vaccine safety with parents. This study contributes to the understanding of under-investigated framing effects of parents vaccinating their children against COVID-19 and have important implications for promoting COVID-19 vaccination in children in the future.

The results of our study should be interpreted in light of its limitations. First, our study was only conducted in China, a non-Western cultural context. As cultural differences in vaccine-related reactions are common [83–86], the extent to which these results can be generalized to other countries is unknown. Second, we recruited the sample via the internet and the online survey respondents may have contributed to self-selection bias or the disproportionate youth of their samples. Third, there was a limitation in not employing quota sampling to ensure demographic representativeness, which may affect the generalizability of our findings to the broader population of Chinese parents. In addition, considering potential validity concerns related to participant engagement and the possibility of distractions in the task, enhanced validation techniques can be explored to further strengthen the reliability of online survey data in future work [87]. Finally, this is a cross-sectional study and cannot take into account the possible effects of time, while vaccine attitudes have been shown to be potentially dynamic and changing [88–90].

More work is needed to demonstrate the effects caused by these subtle framing changes. In addition to exploring the framing effects of COVID-19 vaccine safety information, as in this study, more studies should examine the effects of different information content and message delivery formats. Also, it may be worthwhile to consider examining the effects of presenting vaccine information in mixed (positive and negative) frames. Furthermore, future research could examine framing effects among people in different cultural contexts, given that framing effects are not specific to China and have been documented in other countries for other aspects of vaccines [60, 91, 92]. Finally, the study of framing effects involves legal, ethical, and political domains in future research, and larger and more comprehensive studies are needed.

## Supporting information

S1 File (DOCX)

S1 Data (CSV)
