# Supplementary material for: Effects of COVID-19 vaccine safety framing on parental reactions
Source: PLoS One. 2024 Apr 16;19(4):e0302233. doi: 10.1371/journal.pone.0302233 (PMC11020397; doi:10.1371/journal.pone.0302233)
Supplement: S1 File — (DOCX) [file pone.0302233.s001.docx]

Supporting information for

Effects of COVID-19 vaccine safety framing on parental reactions

**This file includes:**

Methods

S1 Table. Results of univariate analysis of MANCOVA models for parental reactions.

S2-S5 Table. Two-sided independent sample t test results of framing effects for the subgroups –gender, education, income and children age.

Methods

Sampling

Our survey was conducted among a random sample of parents across 29 districts in mainland China. 6242 participants were randomly assigned to receive information on the safety of COVID-19 vaccines in the positive(N=3151) or negative(N=3092) frame. The randomization was done using the research website Sojump to place a certain number of questionnaire response invitations on the platform every day, with half assigned to the positive frame group and the other half to the negative frame group, and the system also excluded duplicate fills.

In both groups, eligible parents had to be at least 18 years old, resident of China and at least one child under 18 years of age. Overall, 3861 surveys were completed. Among the full sample, 1978 (51.2%) participants received positively-framed baseline information and 1883 participants (48.8%) received negatively-framed baseline information.

We cannot directly test whether the randomization indeed created similar groups, but we can compare parents in two groups along their baseline characteristics to check that at least their observed characteristics are on sampling distribution similar. The two sample groups were not different in terms of gender (X2(1) = 0.304, p = 0.581), children’s age (X2(2) =1.398, p = 0.497), education (X2(3) =3.365, p = 0.339) and income (X2(2) =1.686, p = 0.430).

Survey experiment design

In our study, participants were required to read their assigned information condition and, therefore, could not be masked to the fact they were given information. Participants completed the self-report outcome measures online, so the research team can be considered as masked in relation to outcome assessments. The research team had no contact with research participants.

We provided respondents with different presentations on COVID-19 vaccine safety to test how parents would react to different framings of COVID-19 vaccine information, and the information is the latest national side effects data on COVID-19 vaccination officially released by China since the launch of large-scale population vaccination work on December 15, 2020. We designed the treatments on the basis of existing theories that vaccine safety information could be salient to public reaction to COVID-19 vaccine and that slight changes in the way information presented can impacts how people perceive and react to it.

S1 Table.

Results of univariate analysis of MANCOVA models for parental reactions.

| **Model** | **F (1,3584)** | ***P* values** |
| --- | --- | --- |
| **Communication** |  |  |
| Intercept | 612.92 | <0.001 |
| Framing | 0.38 | 0.537 |
| Gender | 1.24 | 0.266 |
| Education | 3.25 | 0.071 |
| Income | 5.21 | 0.023 |
| Children age | 0.02 | 0.903 |
| Mood | 26.60 | <0.001 |
| **Risk perception** |  |  |
| Intercept | 985.73 | <0.001 |
| Framing | 0.10 | 0.757 |
| Gender | 1.87 | 0.172 |
| Education | 6.56 | 0.01 |
| Income | 20.53 | <0.001 |
| Children age | 9.14 | 0.003 |
| Mood | 377.85 | <0.001 |
| **Trust** |  |  |
| Intercept | 961.87 | <0.001 |
| Framing | 2.59 | 0.108 |
| Gender | 2.71 | 0.1 |
| Education | 6.39 | 0.012 |
| Income | 9.26 | 0.002 |
| Children age | 0.82 | 0.365 |
| Mood | 412.00 | <0.001 |
| **Involvement - policy support** |  |  |
| Intercept | 875.56 | <0.001 |
| Framing | 4.06 | 0.044 |
| Gender | 0.07 | 0.796 |
| Education | 22.45 | <0.001 |
| Income | 0.11 | 0.736 |
| Children age | 2.51 | 0.113 |
| Mood | 11.31 | 0.001 |
| **Involvement - government priorities** |  |  |
| Intercept | 1144.34 | <0.001 |
| Framing | 10.28 | 0.001 |
| Gender | 38.44 | <0.001 |
| Education | 4.75 | 0.029 |
| Income | 8.02 | 0.005 |
| Children age | 0.19 | 0.663 |
| Mood | 109.86 | <0.001 |
| **Behavioral intention - when available** |  |  |
| Intercept | 967.79 | <0.001 |
| Framing | 0.38 | 0.538 |
| Gender | 0.29 | 0.593 |
| Education | 3.84 | 0.05 |
| Income | 7.18 | 0.007 |
| Children age | 8.39 | 0.004 |
| Mood | 250.34 | <0.001 |
| **Behavioral intention - regular vaccination** |  |  |
| Intercept | 781.66 | <0.001 |
| Framing | 1.41 | 0.235 |
| Gender | 0.61 | 0.437 |
| Education | 12.02 | 0.001 |
| Income | 2.69 | 0.101 |
| Children age | 17.48 | <0.001 |
| Mood | 323.15 | <0.001 |

S2 Table.

Two-sided independent sample t test results of framing effects for the subgroups – gender.

| **Construct** | **Frame** | **N** | **Mean (s.d.)** | ***p*** | **Effect size (r)** |
| --- | --- | --- | --- | --- | --- |
| **Gender: male** |  |  |  |  |  |
| Communication | positive | 746 | 3.28 (1.120) | 0.607 | 0.014 |
|  | negative | 694 | 3.25 (1.104) |  |  |
| Risk perception | positive | 746 | 2.36 (0.969) | 0.778 | 0.007 |
|  | negative | 694 | 2.38 (0.989) |  |  |
| Trust | positive | 746 | 4.12 (0.923) | 0.917 | 0.003 |
|  | negative | 694 | 4.13 (0.874) |  |  |
| Involvement - policy support | positive | 746 | 3.86 (1.044) | 0.134 | 0.040 |
|  | negative | 694 | 3.77 (1.088) |  |  |
| Involvement - government priorities | positive | 746 | 3.48 (0.707) | 0.035 | 0.056 |
|  | negative | 694 | 3.40 (0.747) |  |  |
| Behavioral intention - when available | positive | 746 | 4.28 (0.993) | 0.987 | 0.000 |
|  | negative | 694 | 4.28 (0.937) |  |  |
| Behavioral intention - regular vaccination | positive | 746 | 4.14 (0.960) | 0.211 | 0.033 |
|  | negative | 694 | 4.08 (1.031) |  |  |
| **Gender: female** |  |  |  |  |  |
| Communication | positive | 1232 | 3.31 (1.081) | 0.761 | 0.006 |
|  | negative | 1189 | 3.29 (1.076) |  |  |
| Risk perception | positive | 1232 | 2.44 (0.937) | 0.848 | 0.004 |
|  | negative | 1189 | 2.45 (0.940) |  |  |
| Trust | positive | 1232 | 4.09 (0.880) | 0.038 | 0.042 |
|  | negative | 1189 | 4.02 (0.931) |  |  |
| Involvement - policy support | positive | 1232 | 3.84 (1.010) | 0.219 | 0.025 |
|  | negative | 1189 | 3.79 (1.002) |  |  |
| Involvement - government priorities | positive | 1232 | 3.31 (0.721) | 0.022 | 0.046 |
|  | negative | 1189 | 3.25 (0.739) |  |  |
| Behavioral intention - when available | positive | 1232 | 4.28 (0.911) | 0.421 | 0.016 |
|  | negative | 1189 | 4.25 (0.926) |  |  |
| Behavioral intention - regular vaccination | positive | 1232 | 4.12 (0.984) | 0.600 | 0.011 |
|  | negative | 1189 | 4.09 (0.992) |  |  |

S3 Table.

Two-sided independent sample t test results of framing effects for the subgroups – education.

| **Construct** | **Frame** | **N** | **Mean (s.d.)** | ***p*** | **Effect size (r)** |
| --- | --- | --- | --- | --- | --- |
| **Education: high school and below** |  |  |  |  |  |
| Communication | positive | 103 | 3.14 (1.207) | 0.575 | 0.042 |
|  | negative | 76 | 3.04 (1.168) |  |  |
| Risk perception | positive | 103 | 2.37 (0.992) | 0.530 | 0.047 |
|  | negative | 76 | 2.46 (1.005) |  |  |
| Trust | positive | 103 | 4.09 (0.930) | 0.612 | 0.038 |
|  | negative | 76 | 4.01 (1.013) |  |  |
| Involvement - policy support | positive | 103 | 3.57 (1.151) | 0.794 | 0.020 |
|  | negative | 76 | 3.53 (1.205) |  |  |
| Involvement - government priorities | positive | 103 | 3.23 (0.757) | 0.770 | 0.022 |
|  | negative | 76 | 3.20 (0.864) |  |  |
| Behavioral intention - when available | positive | 103 | 4.37 (0.863) | 0.449 | 0.057 |
|  | negative | 76 | 4.26 (0.998) |  |  |
| Behavioral intention - regular vaccination | positive | 103 | 4.28 (0.974) | 0.576 | 0.042 |
|  | negative | 76 | 4.20 (1.020) |  |  |
| **Education:** **junior college degree** |  |  |  |  |  |
| Communication | positive | 228 | 3.18 (1.096) | 0.922 | 0.005 |
|  | negative | 208 | 3.19 (1.143) |  |  |
| Risk perception | positive | 228 | 2.34 (0.933) | 0.714 | 0.018 |
|  | negative | 208 | 2.38 (0.938) |  |  |
| Trust | positive | 228 | 4.18 (0.878) | 0.042 | 0.097 |
|  | negative | 208 | 4.00 (0.922) |  |  |
| Involvement - policy support | positive | 228 | 3.70 (1.082) | 0.454 | 0.036 |
|  | negative | 208 | 3.63 (1.051) |  |  |
| Involvement - government priorities | positive | 228 | 3.36 (0.753) | 0.124 | 0.074 |
|  | negative | 208 | 3.25 (0.789) |  |  |
| Behavioral intention - when available | positive | 228 | 4.23 (1.041) | 0.983 | 0.001 |
|  | negative | 208 | 4.23 (0.974) |  |  |
| Behavioral intention - regular vaccination | positive | 228 | 4.17 (0.916) | 0.269 | 0.055 |
|  | negative | 208 | 4.06 (1.112) |  |  |
| **Education: undergraduate degree** |  |  |  |  |  |
| Communication | positive | 1502 | 3.33 (1.072) | 0.409 | 0.015 |
|  | negative | 1458 | 3.30 (1.070) |  |  |
| Risk perception | positive | 1502 | 2.43 (0.943) | 0.960 | 0.001 |
|  | negative | 1458 | 2.43 (0.960) |  |  |
| Trust | positive | 1502 | 4.11 (0.884) | 0.232 | 0.022 |
|  | negative | 1458 | 4.07 (0.895) |  |  |
| Involvement - policy support | positive | 1502 | 3.87 (0.994) | 0.104 | 0.030 |
|  | negative | 1458 | 3.81 (1.014) |  |  |
| Involvement - government priorities | positive | 1502 | 3.38 (0.709) | 0.007 | 0.049 |
|  | negative | 1458 | 3.31 (0.725) |  |  |
| Behavioral intention - when available | positive | 1502 | 4.30 (0.911) | 0.553 | 0.011 |
|  | negative | 1458 | 4.28 (0.899) |  |  |
| Behavioral intention - regular vaccination | positive | 1502 | 4.13 (0.956) | 0.295 | 0.019 |
|  | negative | 1458 | 4.10 (0.975) |  |  |
| **Education: postgraduate and above** |  |  |  |  |  |
| Communication | positive | 145 | 3.21 (1.231) | 0.563 | 0.034 |
|  | negative | 141 | 3.29 (1.110) |  |  |
| Risk perception | positive | 145 | 2.40 (1.015) | 0.689 | 0.024 |
|  | negative | 141 | 2.45 (0.960) |  |  |
| Trust | positive | 145 | 3.90 (1.002) | 0.489 | 0.041 |
|  | negative | 141 | 3.99 (1.007) |  |  |
| Involvement - policy support | positive | 145 | 4.01 (1.074) | 0.268 | 0.066 |
|  | negative | 141 | 3.87 (1.081) |  |  |
| Involvement - government priorities | positive | 145 | 3.49 (0.737) | 0.313 | 0.060 |
|  | negative | 141 | 3.40 (0.810) |  |  |
| Behavioral intention - when available | positive | 145 | 4.14 (1.128) | 0.850 | 0.011 |
|  | negative | 141 | 4.16 (1.119) |  |  |
| Behavioral intention - regular vaccination | positive | 145 | 3.87 (1.203) | 0.403 | 0.050 |
|  | negative | 141 | 3.99 (1.153) |  |  |

S4 Table.

Two-sided independent sample t test results of framing effects for the subgroups – income.

| **Construct** | **Frame** | **N** | **Mean (s.d.)** | ***p*** | **Effect size (r)** |
| --- | --- | --- | --- | --- | --- |
| **Income: low level** |  |  |  |  |  |
| Communication | positive | 430 | 3.24 (1.102) | 0.430 | 0.028 |
|  | negative | 389 | 3.18 (1.108) |  |  |
| Risk perception | positive | 430 | 2.54 (0.956) | 0.733 | 0.012 |
|  | negative | 389 | 2.57 (0.967) |  |  |
| Trust | positive | 430 | 4.09 (0.850) | 0.016 | 0.084 |
|  | negative | 389 | 3.94 (0.935) |  |  |
| Involvement - policy support | positive | 430 | 3.81 (0.956) | 0.059 | 0.067 |
|  | negative | 389 | 3.68 (1.046) |  |  |
| Involvement - government priorities | positive | 430 | 3.30 (0.734) | 0.041 | 0.072 |
|  | negative | 389 | 3.20 (0.756) |  |  |
| Behavioral intention - when available | positive | 430 | 4.28 (0.925) | 0.066 | 0.064 |
|  | negative | 389 | 4.15 (1.051) |  |  |
| Behavioral intention - regular vaccination | positive | 430 | 4.14 (0.951) | 0.136 | 0.054 |
|  | negative | 389 | 4.03 (1.111) |  |  |
| **Income: middle level** |  |  |  |  |  |
| Communication | positive | 1043 | 3.29 (1.089) | 0.922 | 0.002 |
|  | negative | 1032 | 3.28 (1.076) |  |  |
| Risk perception | positive | 1043 | 2.38 (0.930) | 0.363 | 0.020 |
|  | negative | 1032 | 2.41 (0.953) |  |  |
| Trust | positive | 1043 | 4.10 (0.911) | 0.358 | 0.020 |
|  | negative | 1032 | 4.06 (0.911) |  |  |
| Involvement - policy support | positive | 1043 | 3.84 (1.043) | 0.722 | 0.008 |
|  | negative | 1032 | 3.83 (1.034) |  |  |
| Involvement - government priorities | positive | 1043 | 3.38 (0.714) | 0.010 | 0.057 |
|  | negative | 1032 | 3.30 (0.753) |  |  |
| Behavioral intention - when available | positive | 1043 | 4.28 (0.941) | 0.721 | 0.008 |
|  | negative | 1032 | 4.26 (0.909) |  |  |
| Behavioral intention - regular vaccination | positive | 1043 | 4.11 (0.973) | 0.640 | 0.010 |
|  | negative | 1032 | 4.09 (0.989) |  |  |
| **Income: high level** |  |  |  |  |  |
| Communication | positive | 505 | 3.36 (1.103) | 0.817 | 0.007 |
|  | negative | 462 | 3.34 (1.087) |  |  |
| Risk perception | positive | 505 | 2.37 (0.976) | 0.398 | 0.027 |
|  | negative | 462 | 2.32 (0.952) |  |  |
| Trust | positive | 505 | 4.13 (0.904) | 0.743 | 0.011 |
|  | negative | 462 | 4.15 (0.883) |  |  |
| Involvement - policy support | positive | 505 | 3.89 (1.035) | 0.105 | 0.052 |
|  | negative | 462 | 3.79 (1.020) |  |  |
| Involvement - government priorities | positive | 505 | 3.43 (0.715) | 0.553 | 0.019 |
|  | negative | 462 | 3.40 (0.705) |  |  |
| Behavioral intention - when available | positive | 505 | 4.30 (0.961) | 0.269 | 0.036 |
|  | negative | 462 | 4.36 (0.855) |  |  |
| Behavioral intention - regular vaccination | positive | 505 | 4.14 (0.999) | 0.811 | 0.008 |
|  | negative | 462 | 4.13 (0.953) |  |  |

S5 Table.

Two-sided independent sample t test results of framing effects for the subgroups – children age.

| **Construct** | **Frame** | **N** | **Mean (s.d.)** | ***p*** | **Effect size (r)** |
| --- | --- | --- | --- | --- | --- |
| **Children age: under 3 years old** |  |  |  |  |  |
| Communication | positive | 532 | 3.28 (1.065) | 0.832 | 0.007 |
|  | negative | 493 | 3.27 (1.102) |  |  |
| Risk perception | positive | 532 | 2.48 (0.874) | 0.525 | 0.020 |
|  | negative | 493 | 2.52 (0.936) |  |  |
| Trust | positive | 532 | 4.10 (0.855) | 0.198 | 0.040 |
|  | negative | 493 | 4.02 (0.924) |  |  |
| Involvement - policy support | positive | 532 | 3.86 (0.998) | 0.575 | 0.018 |
|  | negative | 493 | 3.90 (0.930) |  |  |
| Involvement - government priorities | positive | 532 | 3.36 (0.723) | 0.168 | 0.043 |
|  | negative | 493 | 3.29 (0.743) |  |  |
| Behavioral intention - when available | positive | 532 | 4.26 (0.933) | 0.141 | 0.046 |
|  | negative | 493 | 4.17 (0.957) |  |  |
| Behavioral intention - regular vaccination | positive | 532 | 4.04 (0.982) | 0.444 | 0.024 |
|  | negative | 493 | 3.99 (1.018) |  |  |
| **Children age: 3-11 years old** |  |  |  |  |  |
| Communication | positive | 1201 | 3.31 (1.096) | 0.583 | 0.011 |
|  | negative | 1136 | 3.29 (1.079) |  |  |
| Risk perception | positive | 1201 | 2.42 (0.976) | 0.400 | 0.017 |
|  | negative | 1136 | 2.38 (0.955) |  |  |
| Trust | positive | 1201 | 4.10 (0.907) | 0.475 | 0.015 |
|  | negative | 1136 | 4.07 (0.903) |  |  |
| Involvement - policy support | positive | 1201 | 3.84 (1.027) | 0.033 | 0.044 |
|  | negative | 1136 | 3.74 (1.073) |  |  |
| Involvement - government priorities | positive | 1201 | 3.38 (0.713) | 0.020 | 0.048 |
|  | negative | 1136 | 3.31 (0.732) |  |  |
| Behavioral intention - when available | positive | 1201 | 4.28 (0.942) | 0.927 | 0.002 |
|  | negative | 1136 | 4.28 (0.928) |  |  |
| Behavioral intention - regular vaccination | positive | 1201 | 4.12 (0.986) | 0.654 | 0.009 |
|  | negative | 1136 | 4.11 (1.003) |  |  |
| **Children age: above 11 years old** |  |  |  |  |  |
| Communication | positive | 245 | 3.24 (1.159) | 0.995 | 0.000 |
|  | negative | 254 | 3.24 (1.091) |  |  |
| Risk perception | positive | 245 | 2.24 (0.960) | 0.044 | 0.091 |
|  | negative | 254 | 2.42 (1.009) |  |  |
| Trust | positive | 245 | 4.13 (0.932) | 0.275 | 0.049 |
|  | negative | 254 | 4.04 (0.932) |  |  |
| Involvement - policy support | positive | 245 | 3.88 (1.053) | 0.209 | 0.056 |
|  | negative | 254 | 3.76 (1.038) |  |  |
| Involvement - government priorities | positive | 245 | 3.40 (0.744) | 0.094 | 0.075 |
|  | negative | 254 | 3.29 (0.805) |  |  |
| Behavioral intention - when available | positive | 245 | 4.36 (0.964) | 0.934 | 0.004 |
|  | negative | 254 | 4.37 (0.869) |  |  |
| Behavioral intention - regular vaccination | positive | 245 | 4.33 (0.873) | 0.132 | 0.068 |
|  | negative | 254 | 4.20 (0.988) |  |  |
